# Being prepared to evaluate pregnancy PrEP

**DOI:** 10.1002/jia2.25435

**Published:** 2019-12-18

**Authors:** Amy L Slogrove

**Affiliations:** ^1^ Department of Paediatrics and Child Health Faculty of Medicine and Health Sciences Stellenbosch University Worcester South Africa; ^2^ Ukwanda Centre for Rural Health Department of Global Health Faculty of Medicine and Health Sciences Stellenbosch University Worcester South Africa

**Keywords:** HIV pre‐exposure prophylaxis, pregnancy, prevention, children, women, infant, Africa

Pregnant and breastfeeding women in high HIV‐incidence settings are at great risk for HIV‐acquisition and stand to benefit tremendously from HIV pre‐exposure prophylaxis (PrEP) scale‐up 1. The PrEP Implementation in Young women and Adolescents (PrIYA) programme is to be commended for addressing this challenge by integrating PrEP delivery into routine maternal child health and family planning services in 16 clinics in Kisumu County, Kenya. Dettinger and colleagues used data from the PrIYA programme implementation to describe birth (weight and gestational age) and 6‐week growth outcomes of infants born to women with and without pregnancy PrEP exposure, concluding that outcomes did not differ by PrEP exposure 2. However, methodologic limitations of this evaluation challenge interpretation of the comparisons in birth weight, gestational age and 6‐week growth outcomes between PrEP‐exposed and PrEP‐unexposed pregnancies.

Mother‐infant pairs for this cross‐sectional study were identified at routine 6‐week postnatal check‐ups at which time data on the antecedent outcomes of birth weight and gestational age were abstracted from routine care records. Comparisons were made between pregnancy PrEP‐exposed and PrEP‐unexposed infants for the birth outcomes of preterm birth and low birth weight. Due to the uniqueness of the neonatal period, in which morbidity and mortality is higher than any other period of life, this participant selection strategy risks biasing the study sample to a lower‐risk group of infants who were alive and well enough to present to a routine care visit 6‐weeks after birth and thus less likely to have experienced the adverse birth outcomes of interest 3. This bias is evident in Dettinger et al's study, where the prevalence of preterm birth at 6.4% and low birth weight at 2.2%, are far lower than estimates of these outcomes for the general population of Kenyan infants of 12.3% and 11.5% respectively 4, 5. Consequently, very little can be concluded from this study about whether exposure to PrEP in pregnancy has any association with preterm birth and low birth weight. Similarly, the comparison of growth at 6‐weeks of age in this sample is only generalizable to the sub‐group of infants at lowest risk of having any adverse 6‐week growth outcomes. And additionally, as the authors do recognize, enrolment of infants identified at age 6‐weeks precludes any evaluation of still birth and neonatal mortality, two highly relevant perinatal outcomes.

With anticipated wide‐scale PrEP uptake by millions of otherwise healthy pregnant women without HIV, the acceptable threshold for PrEP‐related adverse effects is likely lower among pregnant women without HIV than among those with HIV who are taking antiretroviral therapy for their own health with the additional substantial benefit of perinatal HIV transmission prevention. Going forward if the question in relation to PrEP safety in pregnancy is whether outcomes are equivalent between PrEP‐exposed and PrEP‐unexposed pregnancies, then future studies should be designed and powered as equivalence or non‐inferiority studies. This requires careful thought *a priori*, and perhaps establishing consensus amongst stakeholders, of what the acceptable margin of equivalence in birth and infant outcomes between PrEP‐exposed and PrEP‐unexposed pregnancies is, to avoid concluding there is no difference based on insufficiently sized studies with imprecise estimates and wide confidence intervals 6, 7.

Considering the potential scale of pregnancy PrEP exposure to come in HIV high burden countries, strong evidence of the safety of PrEP during pregnancy is required. This includes from randomized trials powered to determine equivalence in outcomes and avoid biases inherent in retrospective observational or programmatic evaluations, in combination with carefully considered programmatic evaluations that appreciate the uniqueness of the perinatal period and can provide real‐world evidence for the safety of PrEP during pregnancy. As a global community, being prepared to scale‐up PrEP includes being prepared to provide quality evidence of the safety of PrEP in pregnant women and their PrEP‐exposed children.

## Competing interests

The author has no competing interests.

## Authors' contribution

ALS conceived of and wrote the letter.
